# High External K^+^ Concentrations Impair Pi Nutrition, Induce the Phosphate Starvation Response, and Reduce Arsenic Toxicity in Arabidopsis Plants

**DOI:** 10.3390/ijms20092237

**Published:** 2019-05-07

**Authors:** Reyes Ródenas, Vicente Martínez, Manuel Nieves-Cordones, Francisco Rubio

**Affiliations:** Departamento de Nutrición Vegetal. Centro de Edafología y Biología Aplicada del Segura-Consejo Superior de Investigaciones Científicas, Campus de Espinardo, 30100 Murcia, Spain; r.rodenas@cebas.csic.es (R.R.); vicente@cebas.csic.es (V.M.); mncordones@cebas.csic.es (M.N.-C.)

**Keywords:** potassium, phosphorous, arsenic, uptake, phosphate starvation response

## Abstract

Potassium (K^+^) and phosphorous (Pi) are two of the most important nutrients required by plants and there is an interest in studying how they are acquired. Most studies have focused on the characterization of the mechanisms involved in K^+^ and Pi uptake and their distribution within the plants, as well as the regulatory mechanisms involved. Evidence is emerging which points to interactions in the nutrition of different nutrients and to the existence of crosstalk in the signaling cascades regulating their acquisition. However, the interaction between K^+^ and Pi has been scarcely studied. Here we show that high concentrations of K^+^ in the external solution inhibit Pi uptake and impair Pi nutrition in Arabidopsis plants, resulting in the induction of phosphate starvation response (PSR) and the upregulation of genes encoding root phosphate uptake systems. The high K^+^-induced PSR depends on the PHR1 and PHL1 transcription factors that are key pieces of Pi signaling in Arabidopsis. Importantly, high K^+^ reduces arsenic accumulation in plants and its toxic effects. The results presented may help to design strategies to reduce Pi deficiency as well as the accumulation of arsenic in crops.

## 1. Introduction

Nitrogen, potassium, and phosphorous are among the most important macronutrients for plants [1] and they constitute an important part of the fertilizers applied to crops to increase their yield. The demand for food of a growing population has led to important increases in fertilizer application, a practice that will continue to grow in the future. This use of fertilizers results not only in important direct costs of agriculture production but also in socio-economic and environmental costs [2,3]. Several strategies, including agronomic management or the development of more efficient varieties, can be designed to reduce the use of fertilizers. However, all of them require understanding of how plants acquire nutrients from soil solution.

After many years of research, scientists have uncovered the molecular mechanisms involved in nitrogen, potassium, and phosphorous nutrition and their regulation [4,5,6]. Importantly, in addition to identifying specific aspects of the nutrition of a given nutrient, interactions on their nutrition have been described. Thus, common pieces for the regulation of the nutrition of different nutrients have been identified and a crosstalk has been proposed to exist among the signal cascades regulating the acquisition of different nutrients [7,8].

The interaction between K^+^ and Pi nutrition has received little attention [9]. It has been described in Arabidopsis and tomato plants that Pi deficiency reduces low-affinity K^+^ uptake as well as the expression of the genes encoding AKT1-type channels that mediate it [10]. Interestingly, this deficiency induces the genes encoding the high-affinity K^+^ uptake system HAK5, although a specific low-K^+^ signal is required to develop a functional transporter [11]. Different elements involved in the specific regulation of K^+^ and Pi acquisition by roots have been identified. The systems involved in K^+^ uptake are transcriptionally and post-translationally regulated [12,13]. In the case of Pi, a complex regulatory network that includes transcription factors, SPX-domains proteins, non-coding RNA, and microRNAs has been described [14]. In the network of Pi signaling, the PHR1 and PHR1-like transcription factors play a central role to induce the phosphate starvation response (PSR) that activates Pi uptake from the external solution through PHT1 transporters.

An additional and important aspect related to Pi nutrition is the toxicity induced in plants by the presence of arsenic in the soil. Arsenic is a carcinogen that enters the food chain through plants and produces important health problems in vast areas of the world [15,16]. AsO_4_^3−^ (As(V)) is similar to PO_4_^3−^ and As(V) is accumulated into the plant via Pi transporters [16,17]. Therefore, the interest in studying the interaction between K^+^ and Pi nutrition can be extended to As(V), with important implications regarding its toxicity.

To gain more insights into the interactions between K^+^ and Pi nutrition and arsenic toxicity, we studied the effect of different K^+^ and Pi supplies on plant growth and plant K^+^ and Pi nutritional status. We observed that high K^+^ concentrations impaired Pi nutrition, partially induced the response to Pi, and reduced arsenic accumulation in plants and its derived toxic effects.

## 2. Results

### 2.1. High K^+^ Reduced phr1phl1 Mutant Growth

**Figure 1:**
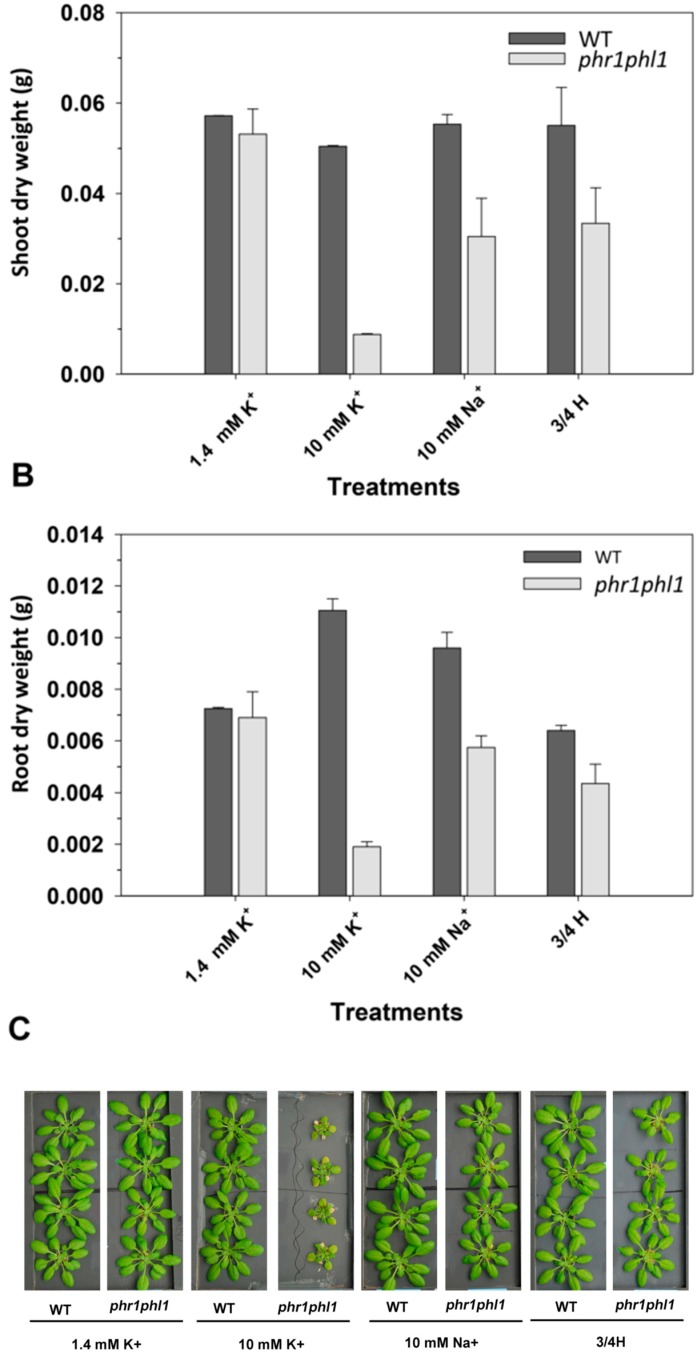
Shoot and root dry weights of plants exposed to 1.4 KCl, 10 mM KCl, 10 mM NaCl, and a concentrated nutrient solution. Plants of WT (dark grey bars) and *phr1phl1* mutant (light grey bars) were grown for 33 days in 1/5 Hoagland control solution and then for 7 days in the presence of 1.4 mM KCl, 10 mM KCl, 10 mM NaCl, and a nutrient concentrated solution (3/4 Hoagland). Plants were separated in shoot and roots, dried at 65 °C, and the dry weights were determined. Shown are average values of shoot (**A**) and roots (**B**) dry weights of three repetitions and errors bars denoted standard error. ANOVA was used to study the effects of the treatment (T), the genotype (G), and their interaction (TxG), resulting in: T = *, G = *** and TxG = ***. Bars with different letters within lines denote significant differences according to Tukey test (uppercase letters for WT and lowercase letters for *phr1phl1* line). Differences between genotypes within the same treatment were analyzed according to ANOVA. ***, **, * indicate significant differences at *p* <0.001, 0.01 and 0.05 respectively and n.s. indicates non-significant at *p* >0.05. (**C**) Picture of plants exposed to the described treatments.

To study the interactions between K^+^ and Pi nutrition, the effect of different K^+^ concentrations on plant growth of WT and *phr1phl1* plants was studied. The *phr1phl1* mutant line lacks the PHR1 and PHL1 transcription factors, which are central pieces of plant response to Pi deficiency [18]. Plants were grown under control conditions (1/5 Hoagland solution) for 33 days and then transferred for 7 days to nutrient solution containing 0.2 mM Pi and two K^+^ levels, 1.4 and 10 mM. It was observed that the presence of 10 mM KCl in the external solution significantly reduced shoot and root growth of *phr1phl1* plants, 84% and 72% respectively (Figure 1A,B). Growth in the presence of 10 mM KCl also reduced growth of WT shoots, although to a much lesser extent, which was not statistically significant (12%) (Figure 1A), and significantly increased WT root growth (Figure 1B).

To study if the KCl-induced growth arrest of the *phr1phl1* plants was specific for K^+^, different treatments were applied. The presence of 10 mM NaCl and a solution with a higher concentration of nutrients (3/4 Hoagland, referred to as 3/4H) with an electrical conductivity (EC) similar to the 10 mM KCl-containing solution were used. These two treatments significantly reduced shoot growth of *phr1phl1* plants (41%). In roots the two treatments reduced growth by 17% and 37% respectively (Figure 1), the latter effect being statistically significant. In WT plants, these treatments had no effect on shoot growth or increased root growth (Figure 1). Determination of shoot/root ratios showed that the 10 mM KCl treatment importantly reduced this parameter in the *phr1phl1* line while increased it in WT plants. The 10 mM NaCl treatment increased the shoot/root ratio in WT and had not effect o *phr1phl1* line and the 3/4H treatment had no effect (Figure S1). The 10 mM NaCl treatment had the same Cl^–^ concentration as the 10 mM KCl treatment, but produced a much smaller effect on *phr1phl1* plant growth. Thus, the NaCl treatment discarded the fact that Cl^–^ was causing the important reduction of growth produced by the 10 mM KCl solution. The high-nutrient treatment (3/4H), with an EC equivalent to that of the 10 mM KCl solution, discarded the fact that the important growth reduction produced by the 10 mM KCl was due to an osmotic effect. Therefore, it could be concluded that the dramatic reduction of *phr1phl1* plant growth because of 10 mM KCl was due to the specific presence of high K^+^. The effects of these treatments on plant growth were visually evident and are presented on Figure 1C.

### 2.2. High K^+^ Reduced Pi Concentrations in phr1phl1 Shoots

**Figure 2:**
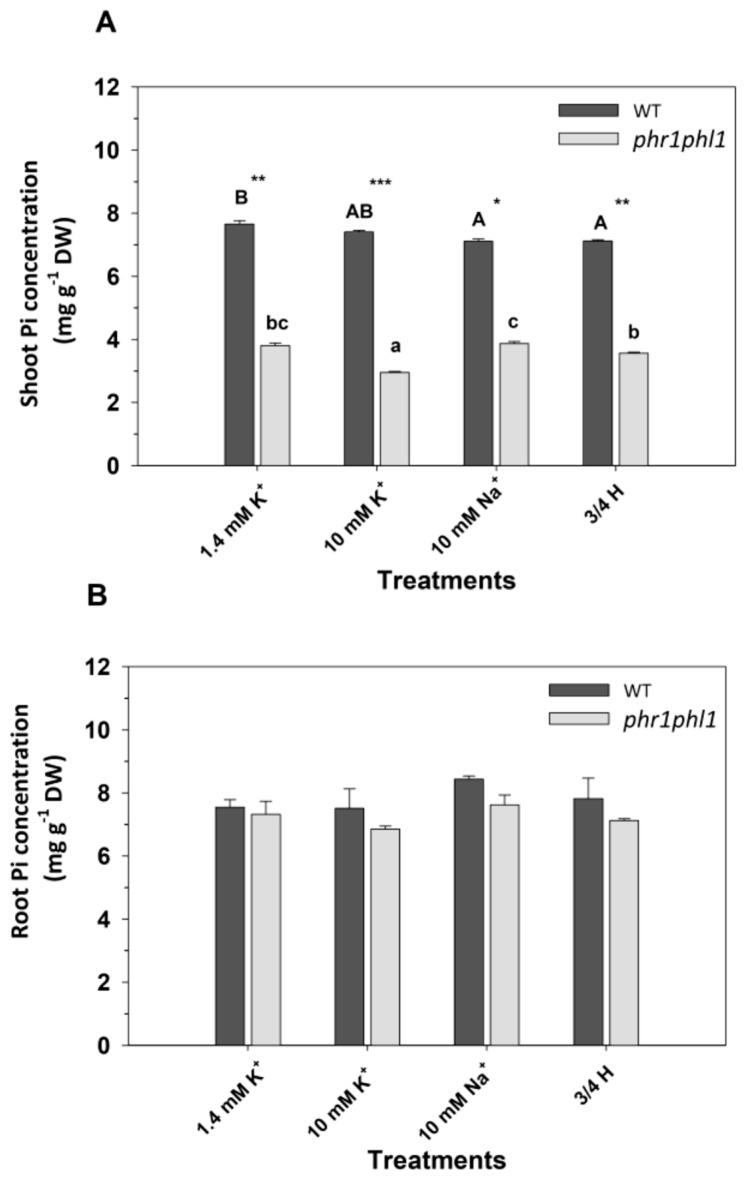
Shoot and root P concentrations of plants exposed to 1.4 mM KCl, 10 mM KCl, 10 mM NaCl, and a concentrated nutrient solution. Plants of WT (dark grey bars) and *phr1phl1* mutant (light grey bars) were grown as described in Figure 1. Collected shoots and roots were dried, acid digested, and their Pi concentrations determined by Inductively Coupled Plasma (ICP) spectrometry analysis. Shown are average shoot (**A**) and root (**B**) Pi concentrations of three repetitions and errors bars denoted standard error. ANOVA was used to study the effects of the treatment (T), the genotype (G), and their interaction (TxG) resulting in: (**A**) T = ***, G = *** and TxG = ***. Bars with different letters within lines denote significant differences according to Tukey test (uppercase letters for WT and lowercase letters for *phr1phl1* line). Differences between genotypes within the same treatment were analyzed according to ANOVA. ***, **, * indicate significant differences at *p* < 0.001, 0.01 and 0.05 respectively. (**B**) T = n.s, G = n.s. and TxG = n.s. n.s indicates non-significant at *p* > 0.05.

Because the *phr1phl1* line is impaired in Pi nutrition, and high K^+^ produced a specific reduction of its growth, the internal Pi and K^+^ concentrations of the plants exposed to the above treatments were determined. It was observed that, in general, *phr1phl1* plants showed significant reduction in their shoot Pi in comparison to WT plants (Figure 2A), consistent with their deficient Pi nutrition [18]. Within *phr1phl1* plants, the 10 mM K^+^ treatment produced a significant decrease in shoot Pi concentration. Shoot Pi concentrations of WT plants were barely affected by the different treatments (Figure 2A). Regarding roots, similar Pi concentrations were found in WT and *phr1phl1* plants. Although it was not statistically significant, the 10 mM K^+^ treatment produced the lowest root Pi concentrations in *phr1phl1* plants (Figure 2B).

When the internal K^+^ concentrations were analyzed, it was observed that, as expected, the plants treated with high K^+^ concentrations showed high organ K^+^ concentrations (Figure S2). In addition, no differences in shoot K^+^ concentrations were found between WT and *phr1phl1* plants (Figure S2A). Both lines showed similar root K^+^ concentrations in the control treatment, but in the rest of the treatments, *phr1phl1* plants showed a reduction of their root K^+^ concentrations with respect to WT (Figure S2B).

**Figure 3:**
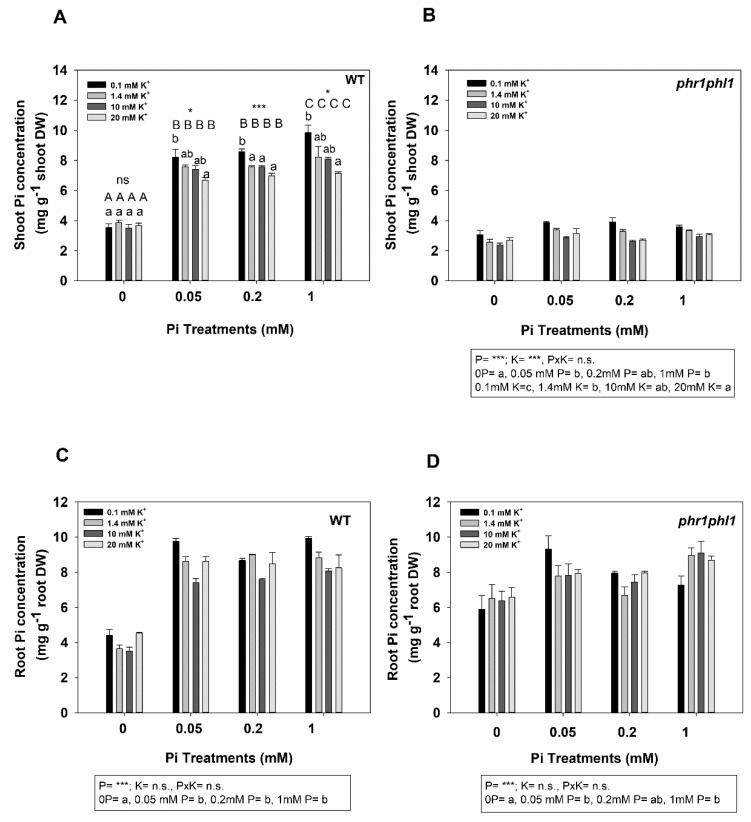
Shoot and root Pi concentrations of plants exposed to different K^+^ and Pi concentrations. Plants of WT and *phr1phl1* mutant were grown for 33 days in a control 1/5 Hoagland solution and the transferred for 7 days to solutions with 0, 0.05, 0.2, or 1 mM Pi and 0.1, 1.4, 10, and 20 mM KCl. After these treatments WT shoots (**A**) and roots (**C**) and *phr1phl1* shoots (**B**) and roots (**D**) were collected, dried, acid digested and their P concentrations determined by ICP spectrometry analysis. Shown are average P concentrations of three repetitions and errors bars denoted standard error. ANOVA was used to study the effects of the Pi treatment (P), the K treatment (K), and their interaction (PxK), resulting in: (**A**) P = ***, K =*** and PxK = *. Bars with different letters within each treatment denote significant differences according to Tukey test (uppercase letters for P treatments and lowercase letters for K treatments). Differences between K treatments within each P treatment were analyzed according to ANOVA. *** and * indicate significant differences at *p* < 0.001 and 0.05 respectively. n.s. indicates non-significant at *p* > 0.05. For (**B**), (**C**) and (**D**) ANOVA and Tukey tests results are indicated below the corresponding panel.

The above results indicated that high external K^+^ concentrations produced reductions of shoot Pi concentrations (Figure 2) and of plant growth in *phr1phl1* plants (Figure 1). An experiment was designed to gain more insight into this K^+^ effect. The idea was to determine if increasing the external Pi concentration could counteract the negative effect of high external K^+^ on Pi concentrations of *phr1phl1* shoots and whether high K^+^ could also affect shoot Pi concentrations of WT plants. Several treatments were applied that included four levels of external K^+^ (0.1, 1.4, 10, and 20 mM K^+^) and four levels of external Pi (0, 0.05, 0.2, and 1 mM). WT plants grown in 0 Pi showed very low shoot Pi concentrations (Figure 3A). These concentrations increased significantly when external Pi was raised from 0 to 0.05 mM and from 0.2 to 1 mM. The effect of external K^+^ depended on the external Pi concentrations. In the absence of external Pi, the K^+^ treatments had no effect. However, for the treatments that contained Pi (0.05, 0.2, and 1 mM), increasing K^+^ significantly decreased the shoot Pi concentration. *phr1phl1* plants showed a much lower shoot Pi concentration than WT plants (Figure 3B). In the absence of external Pi, *phr1phl1* plants showed the lowest Pi concentration. Increasing external Pi from 0 to 0.05 mM, significantly increased shoot Pi concentrations but further increases in Pi to 0.2 and 1 mM did not lead to higher shoot Pi concentrations (Figure 3B). In these plants, increasing external K^+^ significantly reduced shoot Pi concentrations under all conditions of Pi supply. Importantly, *phr1phl1* shoots never increased their shoot Pi concentrations above the levels shown by Pi-starved WT plants, even at the highest external Pi (1 mM) combined with the lowest K^+^ (0.1 mM) concentrations (Figure 3B). In summary, the results showed that high external K^+^ could decrease shoot Pi concentrations of WT plants and that high external Pi did not revert the detrimental effect of high K^+^ on the Pi concentrations of *phr1phl1* shoots.

Regarding roots, increasing external Pi from 0 to 0.05 mM significantly increased root Pi concentrations and further increases had no effect. No effects of external K^+^ were found on the Pi concentrations of the roots of both plant lines (Figure 3C,D). In the absence of external Pi, roots of WT plants showed lower Pi concentrations than those of *phr1phl1* plants. Calculation of the percentage of Pi in shoots showed that, in general, the percentage of Pi in *phr1phl1* shoots was lower than in WT plants (Figure S3). In the presence of 0.05 and higher external Pi, no remarkable differences in root Pi concentrations were found between the two lines (Figure 3C,D).

### 2.3. High External K^+^ Inhibited Root Pi Influx in Pi-Starved Plants

**Figure 4:**
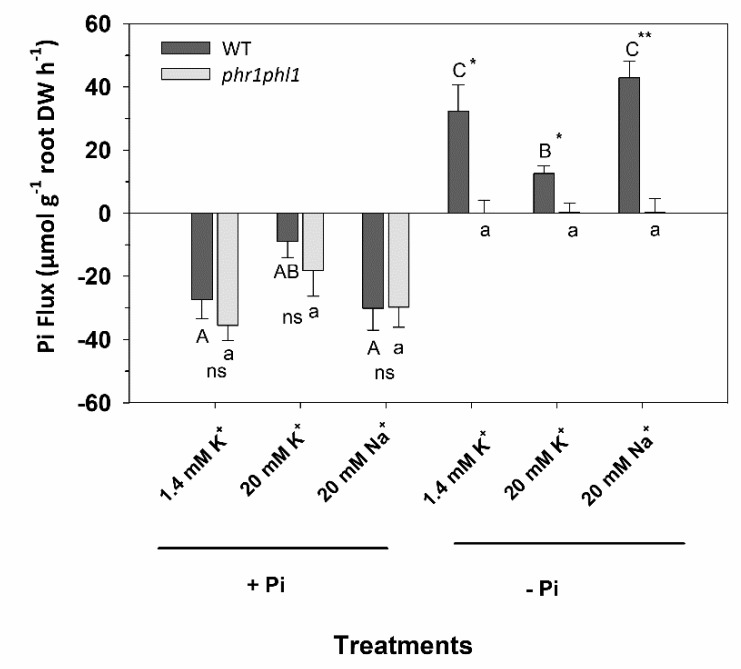
Pi fluxes in Pi-sufficient and Pi-starved plants from a 30 µM external Pi in the absence or the presence of 1.4 mM KCl, 10 mM KCl or NaCl. Plants of WT (dark grey bars) and *phr1phl1* mutant (light grey bars) were grown for 33 days in a control 1/5 Hoagland solution containing 0.2 mM Pi and then transferred for 7 days to the same solution (+Pi) or a solution without Pi (−Pi). Then, plants were incubated for 8 h in nutrient solution with 30 µM Pi in the absence or in the presence of 10 mM KCl or 10 mM NaCl. Samples of this external solution were taken at different time points to determine their Pi concentration. Flux rates of Pi were calculated from the variation in external Pi per h and per g of root dry weight. Shown are average Pi fluxes of three repetitions and errors bars denoted standard error. ANOVA was used to study the effects of the treatment (T), the genotype (G), and their interaction (TxG) resulting in: (A) T = ***, G = *** and TxG = *. Bars with different letters within lines denote significant differences according to Tukey test (uppercase letters for WT and lowercase letters for *phr1phl1* line). Differences between genotypes within the same treatment were analyzed according to ANOVA., **, * indicate significant differences at *p* < 0.01 and 0.05 respectively, n.s. indicates non-significant at *p* > 0.05.

The observed effects of external K^+^ on shoot Pi concentrations (Figure 2 and Figure 3) and plant growth (Figure 1) could be due to an inhibition of Pi uptake by high external K^+^. To study this possibility, plants were grown for 33 days in control 1/5 Hoagland followed by 7 days in solutions with or without Pi. Then, plants were transferred to a solution containing 30 µM Pi in the presence of 1.4 or 20 mM K^+^ or 20 mM Na^+^. The net flux of Pi from the 30 µM solution was determined from the depletion of Pi in the external solution. Pi-grown plants were not able to deplete external Pi, these plants showing an efflux of Pi from the root to the external medium (Figure 4). No significant differences between WT and *phr1phl1* plants were observed. By contrast, Pi-starved WT plants depleted external Pi and, a net flux rate of 32.3 µmol Pi g^−1^ DW h^−1^ could be calculated. When the depletion experiment was performed in the presence of 20 mM K^+^, the flux rate was significantly reduced by 63%, whereas when performed in the presence of 20 mM Na^+^ the flux rated was not affected. *phr1phl1* plants did not show Pi depletion in any of the conditions studied. It could be concluded that high K^+^ concentrations produced a specific inhibition of Pi influx in Pi-starved plants.

### 2.4. High External K^+^ Partially Induced Genes Involved in Pi Starvation Response

**Figure 5:**
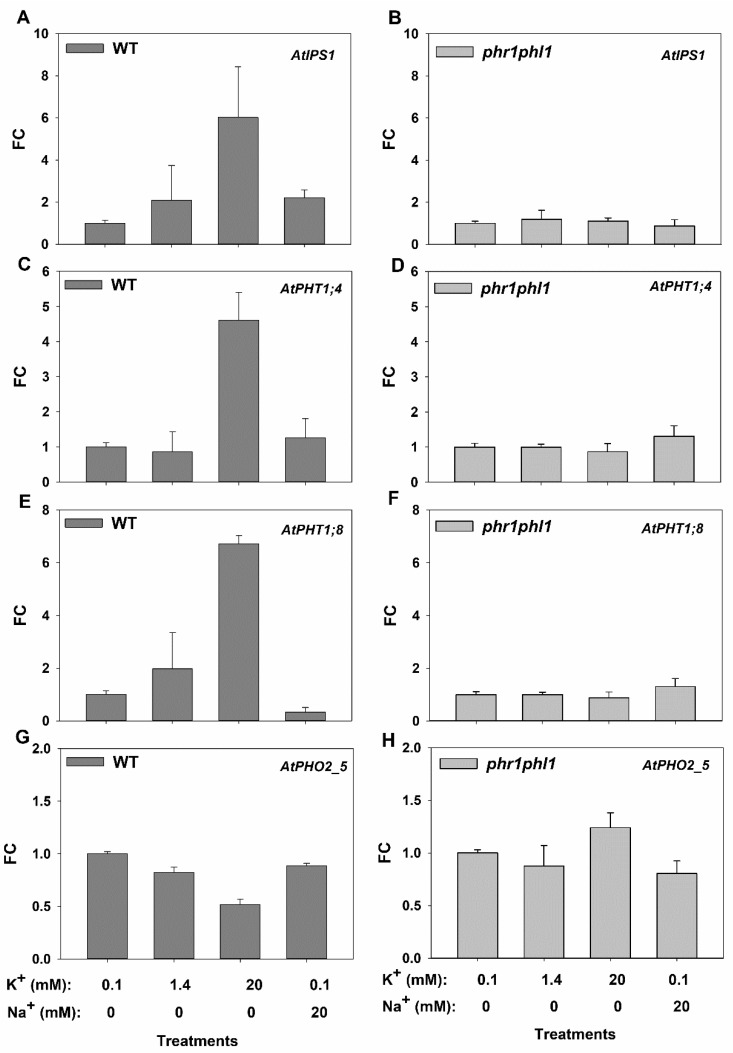
Effects of external K^+^ and Na^+^ on the expression of the *AtIPS1*, *AtPHT1.4*, *AtPHT1.8*, *AtPHO2_5*, *AtPHR,1* and *AtPHL1* genes. Plants of WT (dark grey bars) and *phr1phl1* mutant (light grey bars) were grown for 33 days in a control 1/5 Hoagland solution and then transferred for 7 days to a solution containing 0.05 mM Pi in the presence of 0.1, 1.4, 20 mM KCl, or 0.1 mM KCl + 20 mM NaCl. Then roots were harvested and their total RNA extracted to synthesize cDNA that was used for real time PCR determinations of gene expression by the ∆∆Ct method. (**A**) Expression of *AtIPS1* in WT plants, (**B**) expression of *AtIPS1* in *phr1phl1* plants, (**C**) expression of *AtPHT1;4* in WT plants, (**D**) expression of *AtPHT1;4* in *phr1phl1* plants, (**E**) expression of *AtPHT1;8* in WT plants, (**F**) expression of *AtPHT1;8* in *phr1phl1* plants, (**G**) expression of *AtIPHO2_5* in WT plants and (**H**) expression of *AtIPHO2_5* in *phr1phl1* plants. Shown are the averages of three repetitions for the Fold-Change of gene expression with respect to the calibrator sample corresponding to the 0.1 mM KCl treatment.

**Figure 6:**
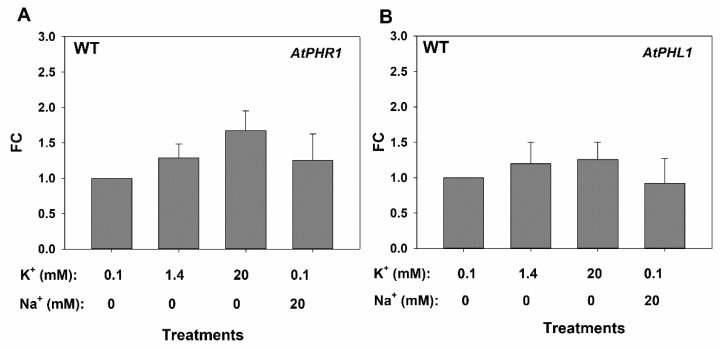
Effects of external K^+^ and Na^+^ on the expression of the *AtPHR1* (**A**) and *AtPHL1* (**B**) genes. Plants of WT were grown and processed as described in Figure 5. Then roots were harvested and their total RNA extracted to synthesize cDNA that was used for real time PCR determinations of gene expression by the ∆∆Ct method. Shown are the averages of three repetitions for the Fold-Change of gene expression with respect to the calibrator sample corresponding to the 0.1 mM KCl treatment.

The effects of high external K^+^ or Na^+^ on the expression of the genes encoding root Pi uptake systems induced by Pi starvation, AtPHT1;4 and AtPH1;8, were studied. The *AtIPS1* gene, encoding a non-coding RNA, and the *PHO2*, encoding an E2 conjugase, were also chosen for this study as they are important pieces of the signal cascade induced by Pi deprivation [14]. Plants were grown in control solution for 33 days and then for 7 days in solutions containing 0.05 mM Pi in the presence of 0.1, 1.4, or 20 mM KCl, or 20 mM Na^+^. After these treatments roots were collected to determine gene expression by real time PCR. In WT plants, 20 mM K^+^ induced 4.8-fold the *AtIPS1* gene (Figure 5 A), 7.6-fold the *AtPHT1.4* gene (Figure 5C), and 8.8-fold the *AtPHT1.8* gene (Figure 5E) with respect to the control treatment (0.1 mM K^+^). By contrast, 20 mM Na^+^ had no effect. These treatments produced no effect on the expression of these genes in the *phr1phl1* background (Figure 5B,D,F). The expression of the *AtPHO2_5* gene was repressed by increasing external K^+^ (Figure 5G) and it was barely affected by the presence of 20 mM Na^+^ in WT plants. In *phr1phl1* plants, *AtPHO2_5* expression was not affected by the treatments (Figure 5H). Finally, the response to 20 mM external K^+^ or Na^+^ of the genes encoding the PHR1 and PHL1 transcription factors was also determined in WT plants. While the expression of *PHL1* (Figure 6B) was not affected by the treatments, increasing external K^+^ slightly increased *PHR1* expression (Figure 6A).

### 2.5. High External K^+^ Reduced Arsenic Toxicity

**Figure 7:**
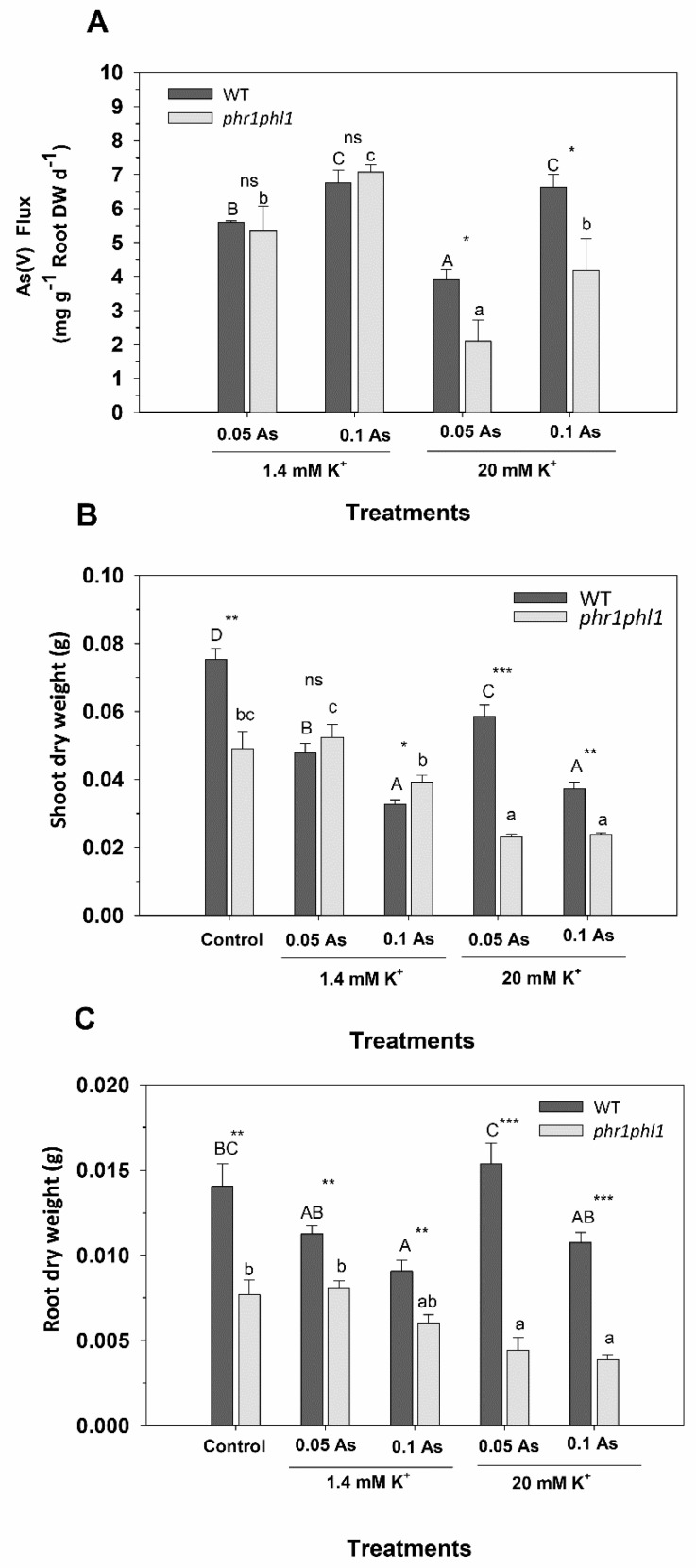
As(V) fluxes and shoots and roots dry weights of plants exposed to different concentrations of As(V) and K^+^. Plants of WT (dark grey bars) and *phr1phl1* mutant (light grey bars) were grown for 33 days in a control 1/5 Hoagland solution containing 0.05 mM Pi and then transferred for 1 day to solutions containing 0.05 mM Pi with 0.05 or 0.1 mM As(V) in the presence of 1.4 or 20 mM K^+^. Then shoots and roots were harvested, dried at 65 °C, acid digested, and their arsenic concentrations determined by ICP spectrometry. The arsenic fluxes (**A**) were calculated from the arsenic accumulation in the plant per day and gram of root dry weight. The average shoot (**B**) and root (**C**) dry weights of these plants are shown. Shown are the averages of three repetitions and error bars denote standard error. ANOVA was used to study the effects of the treatment (T), the genotype (G) and their interaction (TxG), resulting in: (**A**) T = ***, G = * and TxG = *. Bars with different letters within lines denote significant differences according to Tukey test (uppercase letters for WT and lowercase letters for *phr1phl1* line). Differences between genotypes within the same treatment were analyzed according to ANOVA. * indicate significant differences at *p* < 0.05 and n.s. indicates non-significant at *p* > 0.05. (**B**,**C**). T= ***, G = *** and TxG = ***. Bars with different letters within lines denote significant differences according to Tukey test (uppercase letters for WT and lowercase letters for *phr1phl1* line). Differences between genotypes within the same treatment were analyzed according to ANOVA. ***, **, * indicate significant differences at *p* < 0.001, 0.01 and 0.05 respectively and n.s. indicates non-significant at *p* > 0.05.

The above results showed that the presence of high K^+^ concentrations inhibited Pi uptake (Figure 4) and partially induced the Phosphate Starvation Response (PSR) (Figure 5). Since AsO_4_^3−^ (As(V)) is similar to PO_4_^3−^ and it enters the root cells through the Pi transporters, we studied the effect of high K^+^ concentrations on the accumulation of arsenic in the plants from a solution containing As(V). Plants were grown for 33 days in nutrient solution containing 0.05 mM Pi and then for 1 day in solutions that contained 0.05 mM Pi in the presence of 1.4 or 20 mM K^+^ and in the presence of 0, 0.05, or 0.1 mM As(V). Plant material was collected, acid-digested, and analyzed by Inductively Coupled Plasma (ICP) spectrometry to determine its arsenic concentration. From the arsenic accumulated, the As(V) flux was determined. It was observed that the net flux of As(V) from 0.05 µM was inhibited by the presence of 20 mM K^+^ in both lines (Figure 7A). However, this inhibitory effect of K^+^ was only observed in the *ph1phl1* line at 0.1 µM As(V). The accumulation rates of As(V) were similar in WT and *phr1phl1* plants in the presence of 1.4 mM K^+^. The effect of the treatments on arsenic distribution within the plant was also studied and it was observed that, with the exception of the 20 mM K^+^ treatment, which increased the percentage of arsenic accumulated in shoots of WT plants, the rest of the treatments had no effect on this parameter (Figure S4). The bio-concentration factor (BCF) for arsenic was also calculated and it was observed that increasing the external concentration of As(V) reduced the BFC in both lines in the presence of 1.4 mM K^+^, and that the presence of 20 mM K^+^ reduced the BFC (Figure S5).

The effects of As(V) and K^+^ on plant growth were also studied. The presence of 0.05 mM As(V) reduced the dry weight of shoot WT plants (Figure 7B) and an increase of As(V) to 0.1 mM shoot and root dry weights (Figure 7B,C). When the 0.05 mM As(V) treatment was applied together with 20 mM K^+^, the reductions in shoot and root dry weights induced by the presence of As(V) were smaller. This beneficial effect was not observed at 0.1 mM As(V). In *phr1phl1* plants, the presence of 0.05 mM As(V) had no effect, and the presence of 0.1 mM As(V) reduced shoot and root growth, although not significantly (Figure 7B,C). In contrast to what is observed in WT plants, the presence of 20 mM K^+^ did not reduce the inhibition of growth induced by As(V) in the mutant line, on the contrary, the presence of 20 mM K^+^ further decreased shoot and root dry weights of *phr1phl1* plants.

## 3. Discussion

While interactions in the nutrition of NO_3_^–^ and K^+^ [19] and P, S Fe, and Zn [20,21,22,23] have been described, scarce information regarding the effect of K^+^ on Pi nutrition is available. Some studies focused on the interactions between K^+^ and Pi deficiencies in plants [24] and in yeast [25]. In studies on the effect of mycorrhiza on plant nutrition, an interaction between Pi and K^+^ regarding their membrane transport and transfer to the host plant in ectomycorrhizal symbiosis was observed [26]. The present study provides evidence on the negative effect of high external K^+^ concentrations on Pi nutrition in Arabidopsis plants. The study was performed with the WT Col-0 line of Arabidopsis and a *phr1phl1* mutant, defective in plant response to Pi starvation because the genes encoding the PHR1 and PHL1 transcription factors are knocked out [18,23]. *phr1phl1* plants grown under control conditions with a moderate K^+^ supply (1.4 mM K^+^) showed a much lower shoot Pi concentrations than WT plants (Figure 2A) in agreement with previous reports [18,27]. Thus, the use of this mutant line allowed us to study the effects of different levels of K^+^ supply in plants close to the limit of Pi deficiency. The results showed that *phr1phl1* plants were extremely sensitive to the presence of 10 mM KCl (Figure 1). This negative effect of KCl on *phr1phl1* growth was specific to K^+^ because the presence of 10 mM NaCl, or a solution with a higher concentration of nutrients (3/4 H) and a similar EC to the 10 mM KCl solution, but that only contained 1.4 mM KCl, did not reduce *phr1phl1* growth to the levels of the 10 mM KCl solution (Figure 1). An osmotic effect due to a high ion concentration could not be discarded because the 10 mM NaCl and the 3/4H solutions, that lack high K^+^ concentrations, although to a much lesser extent, also reduced plant growth (Figure 1). Thus, it could be concluded that the presence of 10 mM KCl produced a mild osmotic stress to *phr1phl1* plants besides a clear K^+^-specific detrimental effect.

When the shoot Pi concentrations were analyzed, it could be observed that, in comparison with the 10 mM NaCl and the 3/4H treatments, the 10 mM KCl treatment was the only one that reduced shoot Pi concentrations of *phr1phl1* shoots (Figure 2A). Thus, the inhibition of *phr1phl1* plant growth by 10 mM KCl (Figure 1) could be explained by the reduction of its shoot Pi concentration (Figure 2). As reported previously [28], and shown here (Figure 3A), Pi starvation reduces shoot Pi concentration. Our results show that, *phr1phl1* plants never showed a shoot Pi concentration higher than that of Pi-starved WT plants, even in the presence of high Pi concentrations (1mM) (Figure 3B). Importantly, when external K^+^ was increased to 10 or 20 mM KCl, *phr1phl1* shoot Pi concentrations were further reduced (Figure 3B) and plant growth importantly affected (Figure 1). Thus, a threshold in shoot Pi seems to exist below which plant growth is importantly impaired. *phr1phl1* shoots are slightly above this Pi limit under low K^+^ supply (0.1 and 1.4 mM K). The presence of high external K^+^ leads to a reduction in *phr1phl1* shoot Pi concentration. Although this reduction in shoot Pi concentration is not of a great magnitude, it results in shoot Pi concentrations below the mentioned threshold (Figure 3B), leading to an important growth reduction (Figure 1). In WT shoots, with the exception of Pi-starved plants, increasing K^+^ also reduced Pi concentrations, although the mentioned shoot Pi threshold was never reached (Figure 3A). Regarding roots, a clear effect of increasing K^+^ on their Pi concentrations was not observed (Figure 3B). In agreement with previous results, the roots of *phr1phl1* plants starved of Pi showed Pi concentrations higher than those of Pi-starved WT plants (Figure 3B), probably because these plants are not only affected in Pi acquisition but also in their translocation [28]. The impairment in Pi translocation in *phr1phl1* plants is supported by the lower percentage of Pi in the shoots of the mutant line in comparison with WT plants (Figure S3).

The observed effects of high K^+^ on plant Pi concentrations can be explained to some extent by the inhibition of root Pi uptake at the root when the external K^+^ concentrations are high (Figure 4). When WT plants are starved of Pi for 7 days and then transferred to a solution containing 30 µM Pi, a positive net flux of Pi is observed, compatible with the activation of Pi uptake (Figure 4). This net flux is importantly reduced in the presence of 20 mM K^+^ but not in the presence of 20 mM Na^+^. Thus, high K^+^ is specifically inhibiting Pi uptake. The Pi influx observed in the *phr1phl1* mutant was of a very low magnitude (Figure 4), because this line lacks the induction of Phosphate Starvation Response (PSR) and the activation of PHT1 root uptake systems that mediate Pi uptake from the 30 µM solution [14]. Therefore, the effects of high external K^+^ on root Pi uptake could not be studied in this short-term Pi depletion experiments in the *phr1phl1* line. However, the long-term experiments clearly showed that Pi accumulation was reduced in this mutant when external K^+^ was supplied at high concentrations (Figure 2 and Figure 3).

High external K^+^ specifically affected the expression of genes related to the PSR in WT plants (Figure 5). This response is complex and involves transcriptional, post-transcriptional, and post-translational regulation [14]. PHR1 and PHL1 are constitutively expressed transcription factors whose activity is negatively regulated by SPX-domain proteins. Under Pi starvation, PHR1 and PHR-like factors are activated and induce the genes encoding PHT1 Pi root uptake systems, miR399 microRNA, and the *AtIPS1* gene of the non-coding RNA that negatively modulates miR399. This microRNA promotes post-transcriptional inhibition of AtPHO2, an E2-conjugase that mediates negative post-translational control of PHT1 for root Pi uptake [14]. In summary, Pi starvation induces genes encoding root Pi uptake systems and at the same time stabilizes these transport systems. High external K^+^ concentrations, but not Na^+^, induced the *AtIPS1*, *AtPHT1;4*, and *AtPHT1;8* genes (Figure 5), and repressed the *AtPHO2_5* gene in WT plants. This expression pattern is consistent with an induction of the PSR in the plants exposed to high concentrations of K^+^ [14].

Thus, the results presented here show that in the presence of high K^+^, the inhibition of Pi uptake at the root (Figure 4) leads to a reduction in internal Pi concentrations in the plant (Figure 2 and Figure 3), producing a partial Pi starvation that induces genes related to the PSR (Figure 5). One explanation for the observed inhibition of Pi uptake by high K^+^ is that, the plasma membrane depolarization induced by high K^+^ reduces the driving force for Pi uptake through Pi transport systems. These systems mediate accumulation of H_2_PO_4_^–^ in cotransport with H^+^, to overcame the 1000 to 10,000 Pi concentration gradient between the external solution and the cell cytoplasm [17,29]. Thus, their function is highly dependent on the electrical and pH gradients across the plasma membrane. The plasma membrane is highly permeable to K^+^ and, high K^+^ concentrations produce depolarization [30], reducing the driving force for Pi uptake. Because the plasma membrane is more permeable to K^+^ than to Na^+^, the former cation produces a higher depolarization than the latter [31], the inhibition of Pi uptake is more affected by high K^+^ than by high Na^+^ concentrations (Figure 4) and high K^+^ but not Na^+^ induces genes related to PSR (Figure 5).

**Figure 8:**
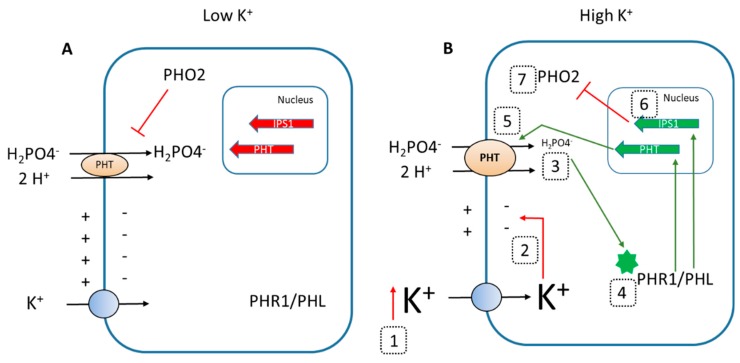
Induction of Phosphate Starvation Respones (PSR) by high external K^+^. The figure shows a simplified proposed model for the mechanism involved in the induction of the PSR by high external K^+^. Shown are key elements of the PSR that have been characterized in the present study. (**A**) Under low K^+^, a non-depolarized membrane potential drives the uptake of H_2_PO4^−^ through the PHT symporter, the cell does not detect Pi deficient, the PHR1 and PHL1 transcription factors are not activated, the transcription of Phosphate Transporters (PHT) is not induced, and the PHO2 conjugase also reduces their activity. (**B**) In the presence of high external K^+^ (1, red upward arrow), K^+^ uptake depolarizes the plasma membrane potential (2, red arrow), reducing the driving force for H_2_PO4^−^ and its accumulation in the cell (3). This partial Pi deficiency activates (green arrow) the PHR1 and PHL1 transcription factors (4) that induce the transcription of the PHT genes encoding Pi uptake systems (5, green arrow) and of the non-coding RNA IPS1 (6, green arrow) that indirectly reduces the accumulation of the PHO2 transcript (7), releasing the negative regulation of PHO2 on PHT transporters. The *PHT* and *IPS1* genes are represented by red wide arrows when not induced and by green wide arrows when induced.

The described induction of the PSR by high K^+^ is mediated by the PHR1 PHL1 transcription factors as the *phr1phl1* mutant line does not show such an induction (Figure 5). Interestingly, the presence of 10 mM K^+^, although it did not affect the expression of PHL1, it slightly induced the expression of *PHR1* in WT plants (Figure 6A). *PHR1* expression has been reported to be independent of the Pi supply [27]. However, a recent study showed regulation of the PHR1 gene by low Pi and in response to auxin [32]. Thus, it is possible that the reduction of Pi concentration of plants grown with high K^+^ (Figure 2 and Figure 3) induces *PHR1* expression (Figure 6) and PSR response. Moreover, auxin concentration in roots is affected by K^+^ [33] while K^+^, but not Na^+^, is required for auxin-promoted root growth [34]. Thus, an auxin-mediated *PHR1* activation by the high K^+^ treatment, leading to PSR induction, cannot be discarded and deserves further investigation. A simplified model of the proposed mechanism involved in the induction of the PSR by high external K^+^ is presented in Figure 8.

The results presented here further support the idea of a crosstalk among the signal cascades regulating the acquisition of different nutrients [7,8] and that the availability of a given nutrient affects the acquisition of others. Common elements of this crosstalk have been described such as the plasma membrane potential [11], reactive oxygen species [7], and hormones [35].

An interesting outcome of the present study is the protective effect of high K^+^ on the toxicity induced by arsenic. Arsenic is a class 1 carcinogen that may enter the food chain through plants. It can cause important health problems, especially in certain areas such as in Asia where arsenic may enter the food chain through contaminated rice [15,16]. Therefore, there is an interest in reducing the accumulation of arsenic in crops. Because arsenic can be present as AsO_4_^3−^ (As(V)) in soils, and As(V) is accumulated in plants via Pi transporters [15,16,36], the effect of high external K^+^ on arsenic accumulation and on As(V)-induced growth reduction was studied. Here we observed that high external K^+^ reduced the accumulation of arsenic in WT and *phr1phl1* plants (Figure 7A). This was consistent with the idea that high K^+^ inhibits Pi uptake (Figure 4) and that As(V) enters the plant through root Pi transporters [15,16,36]. In agreement with these results, an evident reduction of As(V) toxicity by high K^+^ was observed in WT plants exposed to 0.05 mM As(V) (Figure 7B,C). However, high K^+^ did not improve growth of *phr1phl1* grown with As(V) (Figure 7B,C). This could be due to the dramatic detrimental effect that high K^+^ has on *phr1phl1* internal Pi concentration (Figure 2 and Figure 3) and growth (Figure 1) that is added to the toxic effect of arsenic.

One interesting observation is that in the presence of 1.4 mM K^+^, WT and *phr1phl1* plants showed similar accumulation of arsenic at the two levels of As(V) employed (Figure 7A). This can be explained because the PSR is not induced in any of the two lines (Figure 5): in WT plants because these conditions do not produce a reduction in internal Pi and the PSR is not induced (Figure 3 and Figure 5) and in *phr1phl1* plants because knock out of *PHR1* and *PHL1* preclude the induction of the PSR. At high K^+^, a different picture emerged. The presence of 20 mM K^+^ reduced to a greater extent the accumulation of arsenic in *phr1phl1* plants than in WT (Figure 7A). This indicated that different systems mediating As(V) uptake, with different sensitivities to K^+^, are operating in both lines under these conditions. In the presence of 20 mM K^+^, the PHT1 Pi transport systems, that also mediate As(V) uptake [15,16], are induced (Figure 5). However, these systems are not induced in *phr1phl1* plants (Figure 5), which take up As(V) through a different system, probably with a higher sensitivity to 20 mM K^+^. This result with As(V) could also explain why *phr1phl1* plants are so sensitive to high K^+^ (Figure 1): the systems mediating Pi uptake in this line, different to those induced in WT plants, are more sensitive to high K^+^ than those operating in WT plants. The characterization of the systems involved in As(V) accumulation in plants may help to develop low-arsenic crops because reduction in As(V) uptake is proposed as one of the strategies for such a goal. In fact, suppression of high-affinity Pi uptake has been proposed as a mechanism for As(V) tolerance [37] and mutants lacking the Pi transporters PHT1.1 and PHT1.4 show arsenic tolerance [17]. However, this approach would also lead to an impairment of Pi nutrition. Reduction of As(V) accumulation without affecting Pi nutrition may be achieved by increasing the Pi/As selectivity of the systems mediating As(V) uptake [16].

The described effects of high external K^+^ on Pi nutrition and arsenic toxicity in plants may have implications regarding agriculture practices and crop management. The results highlight the relevance of an appropriate balance among the different nutrients applied to crops. It should be considered that moderately high concentrations of K^+^ may impact on Pi nutrition when plants grow under a reduced supply of the latter nutrient. This effect of K^+^ may be more important than that produced by an equivalent concentration of Na^+^, that can be easily found in areas affected by salinization [38]. Regarding As(V) accumulation in crops, it has been proposed that, the competition between Si and As(V) for uptake can be exploited by using Si fertilizers to reduce As(V) accumulation [15]. Similarly, by managing the provision of K^+^ to crops, the accumulation of arsenic in plants could be reduced, as shown in the present study.

In conclusion, the capacity to accumulate Pi in the plant and the sensitivity of plants to As(V) seem to depend not only on the Pi nutrition of the plant, but also on the external concentration of K^+^ and, importantly, on the combined effect of Pi and K^+^ nutrition. The results presented here add more support to the idea that control of PSR involves crosstalk between the signaling pathways of Pi starvation and other nutrients and hormones [14].

## 4. Materials and Methods

### 4.1. Plant Materials and Growth Conditions

Plants of Arabidopsis (*Arabidopsis thaliana)* wild type ecotype Col-0 (WT) and the *phr1-3phl1-2* double mutant [23] were used in this work. Arabidopsis seeds were sterilized and placed in microtubes filled with rockwool on 2 L containers with a modified 1/5-strength Hoagland solution (1/5H) with the following macronutrients: 1.4 mM Ca(NO_3_)_2_ ∙2H_2_O, 0.35 mM MgSO_4_ ∙2H_2_O, 1.4 mM KCl, 0.1 mM Ca(H_2_PO_4_)_2_∙H_2_O, and the following micronutrients: 12.5 µM H_3_BO_3_, 1 µM MnSO_4_, 1 µM ZnSO_4_, 0.5 µM CuSO_4_, 0.1 µM (NH_4_)_6_Mo_7_O_24_, 0.1 µM NiSO_4_ and 10 µM Fe- ethylenediamine-*N*,*N′*-bis(2-hydroxyphenylacetic acid (Fe—EDDHA). The different K^+^ and Pi treatments were obtained by modifying the KCl and Ca(H_2_PO_4_)_2_∙H_2_O concentrations of the solutions. Plants were grown in a controlled-conditions chamber (8/16 h day/night cycle at 150 µmol m^−2^ s^−1^ light, 22 °C and 65% relative humidity). The pH of the nutrient solution was adjusted daily to 5.5 and the solutions were renewed weekly. After 33 days in control nutrient solution, plants were grown for 7 days in nutrient solutions of different compositions to apply the required treatments as indicated in each experiment. Then, different determinations were performed.

### 4.2. Plant Growth and Mineral Composition Determination in Plants

Plants were grown in the control 1/5H solution for 33 days. In one experiment, the specific effect of K^+^ on plant growth and on the internal concentrations of Pi and K^+^ was studied by growing the plants for 7 days in solutions containing 10 mM KCl, 10 mM NaCl or a solution with a higher concentration of nutrients (3/4H) that produced an electrical conductivity (EC) similar to the 10 mM KCl solution. In another experiment, the effect of different supplies of Pi and K^+^ concentrations on the internal concentrations of Pi was studied. For this purpose, plants were grown in 1/5H solution for 33 days and then they were transferred for 7 days to solutions containing different levels of Pi (0, 0.05, 0.2, and 1 mM) in combination with different levels of K^+^ (0.1, 1.4, 10, and 20 mM K^+^). To study the effect of external K^+^ on arsenic accumulation and toxicity, plants were grown for 33 days in 1/5H solution with 0.05 mM Pi, and then for 1 day in the absence of As(V) or in the presence of two levels of As(V) (0.05 and 0.1 mM) in combination with two levels of KCl (1.4 and 20 mM). After exposing the plants to the mentioned treatments in each experiment, they were separated into root and shoot and dried at 65 °C for 4 days. Dry weights were determined, plant material was acid digested with HNO_3_: HClO_4_ (2:1, *v*/*v*) and the mineral concentrations of the plant samples were determined by Inductively Coupled Plasma (ICP) mass spectrometry by using an Iris Intrepid II ICP spectrometer (Thermo Electron Corporation). Bio-concentration factor (BCF) for arsenic was calculated as the ratio between the concentrations of arsenic within the plant and in the external medium.

### 4.3. Pi Depletion from External Solution

Plants were grown for 33 days in 1/5H solution and then for 7 days in the same solution or in the absence of Pi. Individual plants were rinsed with a cold Pi-free solution and transferred to 10 mL tubes containing 1/5H solution containing 30 µM PO_4_^3−^ by adding 15 µM Ca(H_2_PO_4_)_2_∙H_2_O (pH = 5.5) and the same solution supplemented with 20 mM KCl or 20 mM NaCl. Samples of the external solution were taken every 30 min during the first 2 h and then every hour for the next 6 h. Pi concentration in the samples was determined by using the Vanadomolybdophosphoric acid colorimetric method [39] with a Power Wave XS2 spectrophotometer (BioTek, Winooski, VT, USA). The rates of Pi net flux were calculated from the variation in Pi concentration in the external solution per gram of root dry weight (DW) and unit of time. Three repetitions per condition and experiment were performed.

### 4.4. RNA Isolation and Real-Time Quantitative PCR Analysis

Plants were grown for 33 days in 1/5H solution and then transferred for 7 days to solutions containing 0.1, 1.4, or 20 mM KCl or to a solution containing 0.1 mM KCl and 20 mM NaCl. After these treatments, total RNA from roots was isolated using the NucleoSpin^®^ RNA Plant kit (Macherey-Nagel, Düren, Germany) and treated with DNA-free^TM^ (Applied Biosystems/Ambion, Austin, TX, USA). cDNA synthesis was carried out with the High capacity cDNA Reverse Transcription Kit (Applied Biosystems). Real-time PCR was performed in a 7500 Real-time PCR system (Applied Biosystems) and the expression levels of transcripts were calculated by using the relative quantification method [40] and using *AtACT2* as reference gene. The calibrator sample corresponded to the treatment containing 0.1 mM K^+^ plus 0.05 mM Pi. The primers used in this work are listed in Supplemental Table S1.

### 4.5. Statistical Analysis

Analysis of variance was performed with the SPSS v.25 for Windows (Chicago, IL, USA) software. Differences among means were compared by using a Tukey’s Multiple Range test.

## Supplementary Materials

Supplementary materials can be found at https://www.mdpi.com/1422-0067/20/9/2237/s1. References [41,42] are cited in the supplementary materials.
