# Supplementary material for: High External K+ Concentrations Impair Pi Nutrition, Induce the Phosphate Starvation Response, and Reduce Arsenic Toxicity in Arabidopsis Plants
Source: Int J Mol Sci. 2019 May 7;20(9):2237. doi: 10.3390/ijms20092237 (PMC6539835; doi:10.3390/ijms20092237)
Supplement: Supplementary file 1 [file ijms-20-02237-s001.pdf]

Supplementary Material

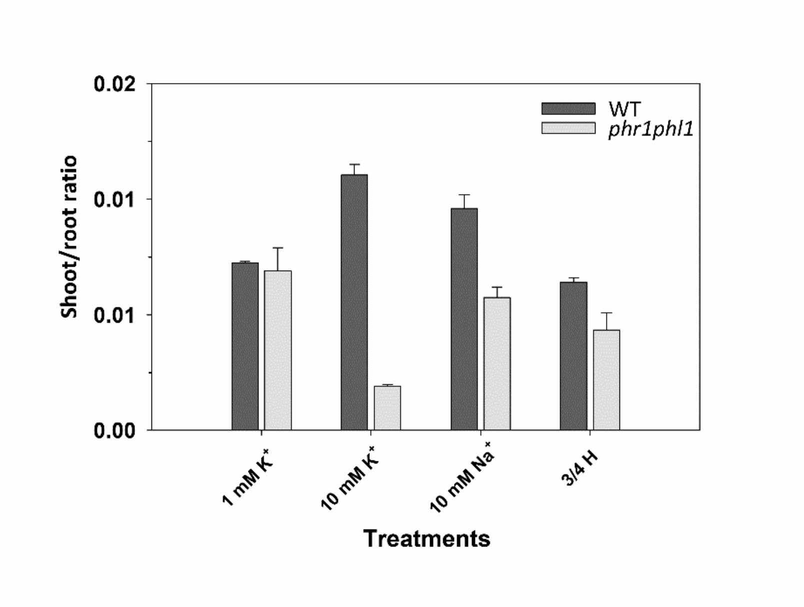

Figure S1. Shoot to root ratio of plants exposed to 1.4 KCl, 10 mM KCl, 10 mM NaCl, and a concentrated nutrient solution. Plants of WT (dark grey bars) and *phr1phl1* mutant (light grey bars) were grown for 33 d in 1/5 Hoagland control solution and then for 7 d in the presence of 1.4 mM KCl, 10 mM KCl, 10 mM NaCl, and a concentrated nutrient solution (3/4 Hoagland), and processed as indicated in Figure 1.

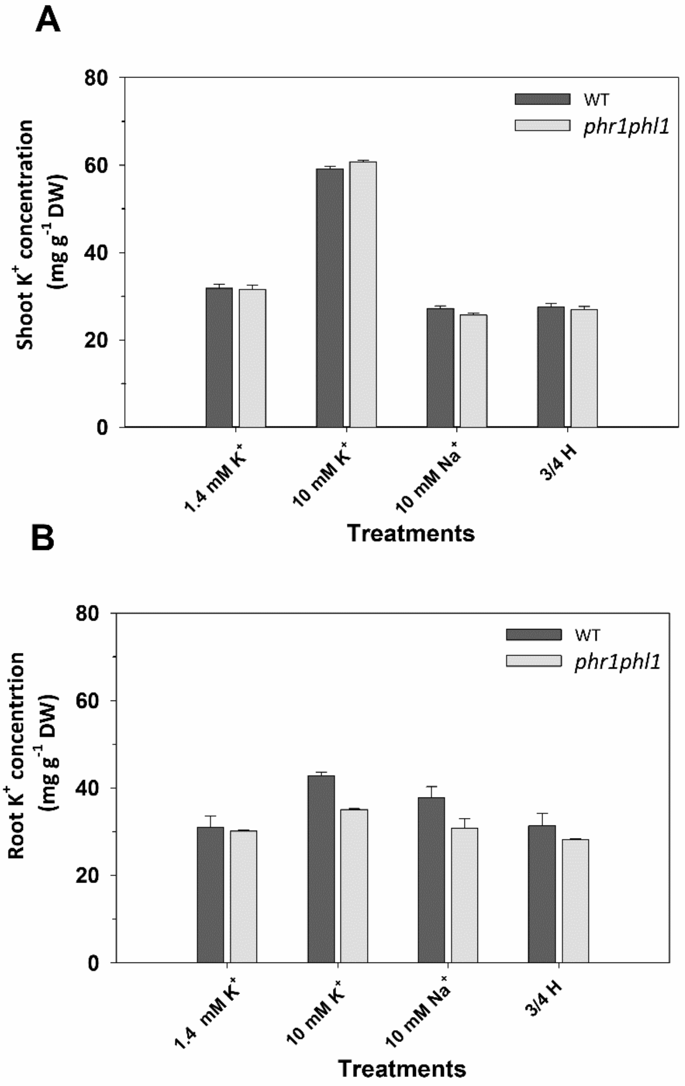

Figure S2. Shoot and root K^+ concentrations of plants exposed to 1.4 mM KCl, 10 mM KCl, 10 mM NaCl and a concentrated nutrient solution. Plants of WT (dark grey bars) and *phr1phl1* mutant (light grey bars) were grown as described in Figure 1. Collected shoots and roots were dried, acid digested, and their K^+ concentrations determined by ICP spectrometry analysis. Shown are average shoot (A) and root (B) K^+ concentrations of three repetitions and errors bars denote standard error.

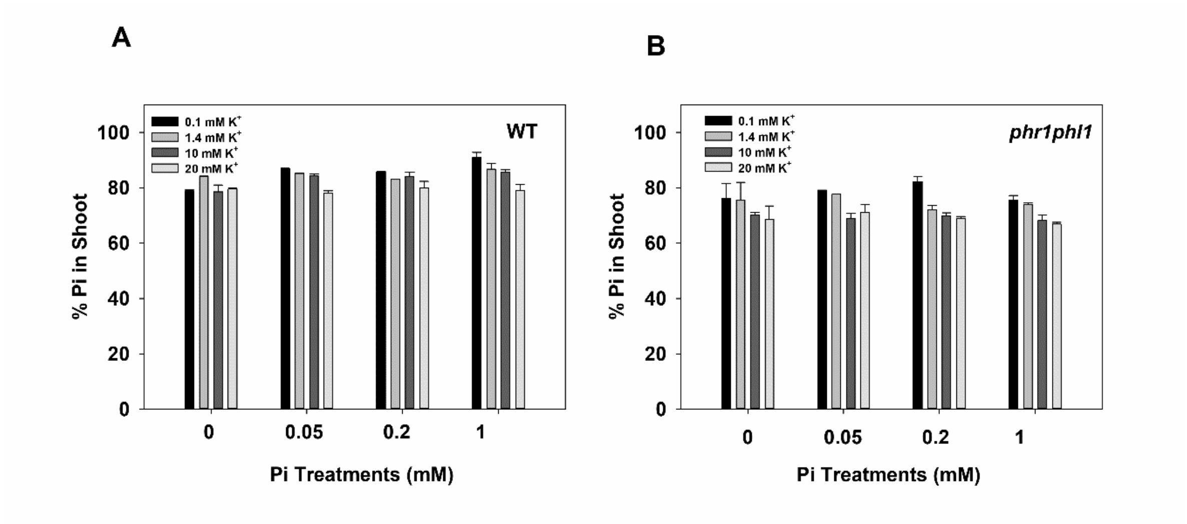

Figure S3. Percentage of Pi in shoots of plants exposed to different K^+ and Pi concentrations. Plants of WT (A) and *phr1phl1* mutant (B) were grown for 33 d in a control 1/5 Hoagland solution and then transferred for 7 d to solutions with 0, 0.05, 0.2, or 1 mM Pi and 0.1, 1.4, 10, and 20 mM KCl. After these treatments plants were processed as indicated in Figure 3.

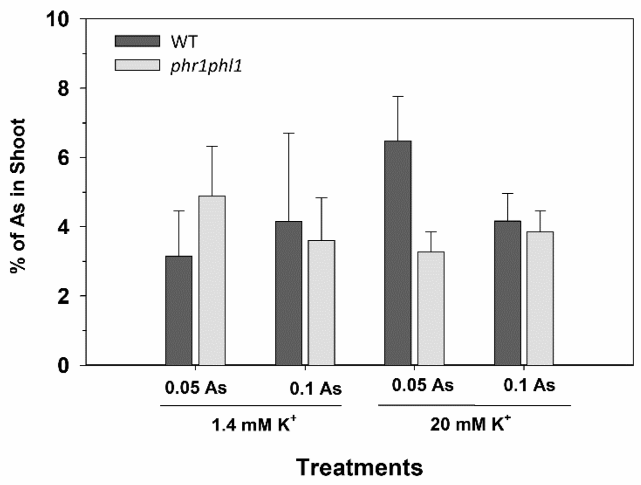

Figure S4. Percentage of arsenic in the shoot of plants exposed to different concentrations of As(V) and K^+. Plants of WT (dark grey bars) and *phr1phl1* mutant (light grey bars) were grown for 33 d in a control 1/5 Hoagland solution containing 0.05 mM Pi and then transferred for 1 d to solutions containing 0.05 mM Pi with 0.05 or 0.1 mM As(V) in the presence of 1.4 or 20 mM K^+. Plants were processed as indicated in Figure 7.

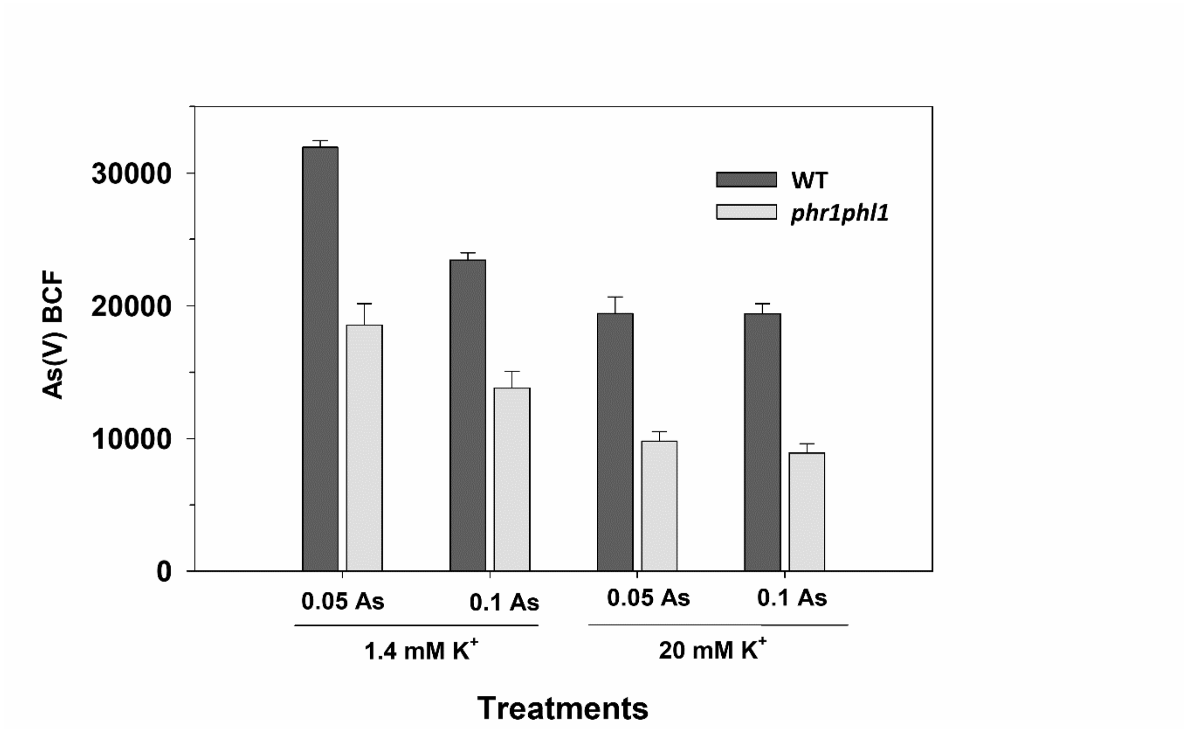

Figure S5. Bio-concentration factor (BFC) of As(V) of plants exposed to different concentrations of As(V) and K^+. Plants of WT (dark grey bars) and *phr1phl1* mutant (light grey bars) were grown for 33 d in a control 1/5 Hoagland solution containing 0.05 mM Pi and then transferred for 1 d to solutions containing 0.05 mM Pi with 0.05 or 0.1 mM As(V) in the presence of 1.4 or 20 mM K^+. Plants were processed as indicated in Figure 7. The Bio-concentrations factor for As(V) was calculated and the ratio between the concentrations of As(V) in the plants and the external concentration of As(V).

Table 1. Primers used for real time qPCR.

Gene	Forward primer (5′-3′)	Reverse primer (5′-3′)	Reference
AtACT2	CGGTGGTTCCATTCTTGCTT	CGGCCTTGGAGATCCACAT	[41]
AtIPS1	AGACTGCAGAAGGCTGATTCAGA	TTGCCCAATTTCTAGAGGGAGA	[42]
AtPHT1;4	TCAATGGCGTTGCCTTCTGT	ATCACCAAGCCACCCGAAA	[42]
AtPHT1;8	ACTGCAGAAAACGTCTACGACG	CAGCGATGATGGCTCCTAATTC	[42]
AtPHO2_5	GTGAAGGACCATTTTACGCACC	CCATATAAGCCTTGCACGCAG	[42]
AtPHR1	TTGGACGCCAGAGCTTCAC	TTCACTACCGCCAAGACTGTTG	This work
AtPHL1	ATGCGTTGGACACCAGAACTT	TCACTACCACCAAGCTGATTAACAG	This work
